# Preterm Labor and Preterm-PROM at a Lower Gestational Age Are Associated with a Longer Latency-to-Delivery Even in Patients with the Same Intensity of Intra-Amniotic Inflammation: “Carroll-Model” Revisited

**DOI:** 10.3390/life12091329

**Published:** 2022-08-27

**Authors:** Jeong-Won Sohn, Eun-Saem Choi, Chan-Wook Park, Kyung-Chul Moon, Joong-Shin Park, Jong-Kwan Jun

**Affiliations:** 1 Department of Obstetrics and Gynecology, Seoul National University College of Medicine, Seoul 03080, Korea; 2 Department of Obstetrics and Gynecology, Korea University College of Medicine, Korea University Anam Hospital, Seoul 02841, Korea; 3 Institute of Reproductive Medicine & Population, Seoul National University Medical Research Center, Seoul 03080, Korea; 4 Department of Pathology, Seoul National University College of Medicine, Seoul 03080, Korea

**Keywords:** intra-amniotic inflammation, gestational age, latency to delivery, preterm labor, preterm-PROM

## Abstract

A previous study by Carroll et al. demonstrated that the time from preterm-PROM to delivery was longer at a lower gestational age (GA) when the membranes rupture, although the presence or absence of intra-amniotic inflammation (IAI) was not examined in that study. However, patients with either preterm labor (PTL) or preterm-PROM at a lower GA had more frequent IAI, which was associated with a shorter amniocentesis-to-delivery (ATD) interval as compared with inflammation-free amniotic fluid (AF). Up to now, there is no information about whether PTL and preterm-PROM at a lower GA are associated with a shorter or longer latency to delivery in cases with the same intensity of IAI. The objective of the study is to examine this issue. AF MMP-8 was measured in 476 singleton early preterm-gestations (21.5 < GA at amniocentesis < 34 wks) with PTL (*n* = 253) and preterm-PROM (*n* = 223). Patients were divided into three groups according to GA at amniocentesis (i.e., group-1: <26 wks; group-2: 26–30 wks; group-3: 30–34 wks). IAI was defined as an elevated AF MMP-8 (≥23 ng/mL), and IAI was classified into either mild IAI (AF MMP-8: 23–350 ng/mL) or severe IAI (AF MMP-8 ≥ 350 ng/mL). ATD interval was examined according to GA at amniocentesis in the context of the same intensity of IAI (i.e., inflammation-free AF, mild IAI, and severe IAI) among pregnant women with either PTL or preterm-PROM. IAI was more frequent at a lower GA in cases with PTL (group-1 vs. group-2 vs. group-3; 59.5% vs. 47.4% vs. 25.1%; *X*^2^ *test, p* = 0.000034 and *linear by linear association [LBLA]*, *p* = 0.000008) and in those with preterm-PROM (group-1 vs. group-2 vs. group-3; 69.2% vs. 50.0% vs. 32.0%; *X*^2^ *test*, *p* = 0.000104, and *LBLA*, *p* = 0.000019). Of note, cases without IAI at a lower GA had a longer ATD interval in both PTL (*Spearman’s rank correlation test*, γ = −0.360, *p* = 0.000003) and preterm-PROM (γ = −0.570, *p* = 0.000001) groups. Moreover, the lower the GA, the longer the ATD interval, even among patients with mild and severe IAI in both PTL (*Spearman’s rank correlation test*; mild IAI, γ = −0.290, *p* = 0.039; severe IAI, γ = −0.299, *p* = 0.048) and preterm-PROM (mild IAI, γ = −0.565, *p* = 0.000013; severe IAI, γ = −0.363, *p* = 0.015) groups. In conclusion, PTL and preterm-PROM at a lower GA are associated with a longer latency to delivery, even in patients with the same intensity of IAI. This finding suggests that a more intense IAI may be needed for spontaneous preterm birth at a lower GA.

## 1. Introduction

Ascending intra-uterine infection and subsequent intra-amniotic inflammation (IAI) are the most important pathophysiologies of spontaneous preterm birth (sPTB) due to either preterm labor and intact membranes (PTL) or preterm premature rupture of membranes (preterm-PROM) [1,2,3]. IAI is present in about 30% of cases with PTL [4,5] and about 40% of cases with preterm-PROM [6,7], respectively. Of note, the lower the gestational age (GA), the higher the frequency of IAI in both PTL and preterm-PROM [4,6]. Moreover, patients with IAI had a significantly shorter amniocentesis-to-delivery (ATD) interval than those with inflammation-free amniotic fluid (AF) [4,6]. Therefore, it is plausible that pregnant women with either PTL or preterm-PROM at a lower GA have a shorter latency to delivery. However, contrary to this expectation, a previous study by Carroll SG et al. demonstrated that the latency from preterm-PROM to delivery was longer at a lower GA when rupture of membranes (ROM) develops, although the presence or absence of IAI was not examined in that study [8]. Unfortunately, the presence or absence and the intensity of IAI should be examined for the analysis of a relationship between GA at the time of either PTL or preterm-PROM and the latency to delivery. However, up to now, there is no information about whether either PTL or preterm-PROM at a lower GA is associated with a shorter or longer latency to delivery in cases with the same intensity of IAI. Notably, we previously demonstrated the higher intensity of IAI was required for the preterm birth at a lower GA even in the same grade of acute chorioamnionitis, leading to the assumption that preterm birth at a lower GA is less frequent in the context of same intensity of IAI [9]. Considering previous reports by Carroll SG [8] and our group [9], it is plausible that either PTL or preterm-PROM at a lower GA is associated with a longer latency to delivery in cases with the same intensity of IAI. Therefore, we hypothesized that either PTL or preterm-PROM at a lower GA would be associated with a longer latency to delivery in cases with the same intensity of IAI. The objective of the study is to examine this issue.

## 2. Materials and Methods

### 2.1. Study Design and Patient Population

The study population included 476 singleton pregnant women who had an amniocentesis due to PTL (253 cases) or preterm-PROM (223 cases) between 21.6 and 33.9 weeks of gestation at Seoul National University Hospital from 26 January 1993 to 28 November 2008, and met the following criteria: (1) documented results of placental pathology; and (2) available results of AF matrix metalloproteinase-8 (MMP-8). At our institution, amniocentesis for the retrieval of AF was routinely offered to all patients who were admitted with the diagnosis of either PTL or preterm-PROM for the identification of intra-amniotic infection or inflammation. Moreover, placental histologic examination was routinely offered and performed for all pregnant women who delivered at preterm due to either PTL or preterm-PROM. PTL and preterm-PROM were diagnosed with previously published criteria [9,10]. Patients were divided into three groups according to GA at amniocentesis (i.e., group-1: <26 weeks; group-2: 26–30 weeks; and group-3: 30–34 weeks). Written informed consent was gained from all study population. The institutional review board of our institute specifically approved the current study (IRB number: 1612-034-812).

### 2.2. Clinical Characteristics and Pregnancy Outcomes

Clinical characteristics and pregnancy outcomes were obtained from a medical record review. Data included maternal age, parity, cause of preterm delivery, GA at delivery, birth weight, gender of newborn, and delivery mode.

### 2.3. Amniotic Fluid (AF)

AF was centrifuged and stored in polypropylene tubes at −70 °C. MMP-8 concentrations in stored AF were measured with a commercially available enzyme-linked immunosorbent assay (Amersham Pharmacia Biotech, Inc., Little Chalfont, UK). The sensitivity of the test was <0.3 ng/mL. Both intra- and inter-assay coefficients of variation were <10%. Details about this assay and its performance were previously described [11]. IAI was defined as the presence of an increased AF MMP-8 concentration (≥23 ng/mL) [6], and IAI was classified into either mild IAI (AF MMP-8: 23–350 ng/mL) or severe IAI (AF MMP-8 ≥ 350 ng/mL). ATD interval was examined according to GA at amniocentesis in the context of the same intensity of IAI (i.e., inflammation-free AF, mild IAI and severe IAI) among pregnant women with either PTL or preterm-PROM.

### 2.4. Diagnosis of Acute Histologic Chorioamnionitis (Acute-HCA) and Funisitis

Placental tissue samples for pathologic examination included extra-placental membranes (i.e., chorio-decidua and amnion), chorionic plate, and umbilical cord. These samples were fixed in 10% neutral buffered formalin and embedded in paraffin. Sections of prepared tissue blocks were stained with hematoxylin and eosin (H & E). Clinical information was not disclosed to pathologists. Acute histologic chorioamnionitis (acute-HCA) was defined as the presence of chorio-deciduitis, amnionitis, or chorionic plate inflammation [12]. Chorio-deciduitis was diagnosed in the presence of a least one focus of >5 neutrophils in the chorio-decidua, amnionitis was diagnosed in the presence of at least one focus of >5 neutrophils in the amnion, and chorionic plate inflammation was diagnosed in the presence of more than one focus of at least ten neutrophilic collections or diffuse inflammation in subchorionic fibrin, or diffuse/dense inflammation, neutrophilic infiltration into connective tissue of placental plate, or placental vasculitis according to the criteria previously published [12]. Funisitis was defined as the presence of the neutrophil infiltration into the umbilical vessel walls or Wharton’s jelly, according to the criteria previously published as well [12].

### 2.5. Statistical Analysis

The Kruskal–Wallis test was used for the comparison of continuous variables. Comparisons of proportions were performed with the Pearson’s chi-square test. The linear-by-linear association test was used for the assessment of trend. Spearman’s rank correlation test was used to investigate the relationship between the amniocentesis-to-delivery interval and the GA at amniocentesis according to the presence or the intensity of IAI in cases with either PTL or preterm-PROM. Data were analyzed using SPSS Statistics 20.0. Statistical significance was defined as *p* < 0.05.

## 3. Results

### 3.1. Clinical Characteristics and Pregnancy Outcomes According to Gestational Age at Amniocentesis in Either Preterm Labor and Intact Membranes (PTL) or Preterm Premature Rupture of Membranes (Preterm-PROM) with the Same Intensity of Intra-Amniotic Inflammation (IAI)

Group-1 (GA at amniocentesis < 26 weeks), group-2 (GA at amniocentesis 26–30 weeks) and group-3 (GA at amniocentesis 30–34 weeks) are present in 16.6% (42/253), 30.0% (76/253) (Table 1), and 53.4% (135/253) of PTL cases, and in 15.4% (39/223), 22.1% (56/223), and 50.6% (128/223) of preterm-PROM cases (Table 2), respectively. GA at delivery was significantly lower according to a lower GA at amniocentesis in the context of mild and severe IAI, while that was not significantly different among the groups in the absence of IAI for both PTL and preterm-PROM cases (Table 1 and Table 2).

### 3.2. Frequency of Intra-Amniotic Inflammation (IAI) and Severe IAI as a Function of Gestational Age (GA) in Cases with PTL and Preterm-PROM

**Figure 1:**
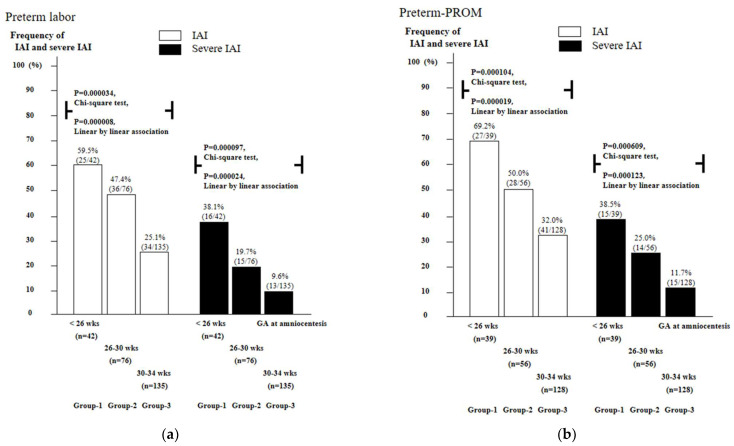
Frequency of intra-amniotic inflammation (IAI) (defined as AF MMP-8 concentration ≥ 23 ng/mL) and severe IAI (defined as AF MMP-8 concentration ≥ 350 ng/mL) as a function of gestational age (GA) in cases with PTL (**a**) and preterm-PROM (**b**). The lower the GA, the higher the frequency of IAI and severe IAI. Frequency and *p*-values are shown. White (open) columns represent IAI, and black (closed) columns represent severe IAI.

Figure 1 shows a lower GA is associated with a significantly higher frequency of IAI (group-1 vs. group-2 vs. group-3: PTL, 59.5% vs. 47.4% vs. 25.1%, *p* = 0.000034 in Chi-square test and *p* = 0.000008 in linear-by-linear association; preterm-PROM, 69.2% vs. 50.0% vs. 32.0%, *p* = 0.000104 in Chi-square test and *p* = 0.000019 in linear-by-linear association) and severe IAI (group-1 vs. group-2 vs. group-3: PTL, 38.1% vs. 19.7% vs. 9.6%, *p* = 0.000097 in Chi-square test and *p* = 0.000024 in linear-by-linear association; preterm-PROM, 38.5% vs. 25.0% vs. 11.7%, *p* = 0.000609 in Chi-square test and *p* = 0.000123 in linear-by-linear association) in both PTL (Figure 1a) and preterm-PROM (Figure 1b).

### 3.3. Frequency of Intra-Amniotic Inflammation (IAI) and Severe IAI as a Function of Gestational Age (GA) in Cases with PTL and Preterm-PROM

**Figure 2:**
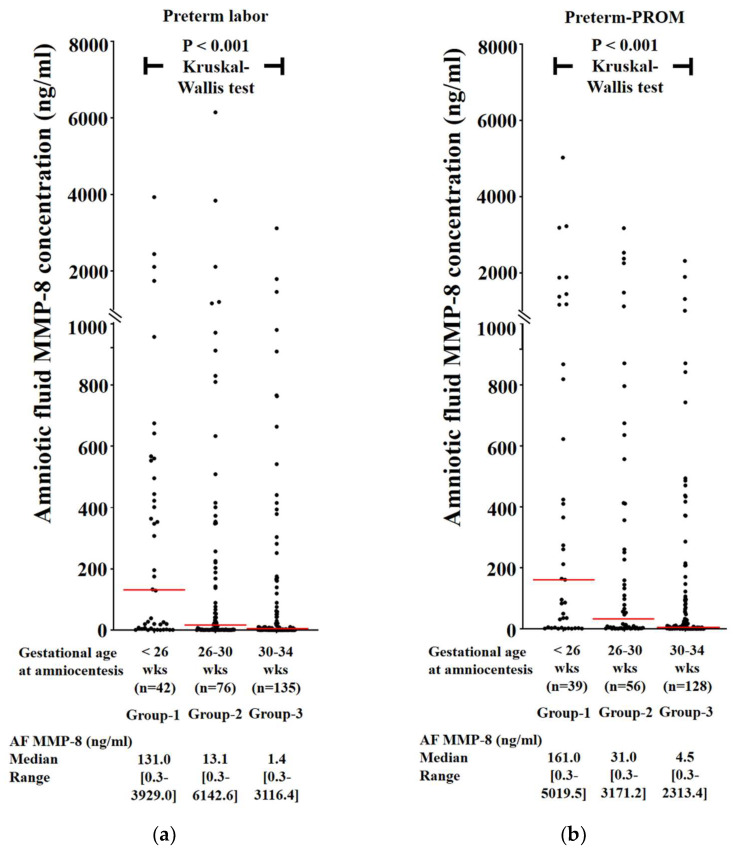
AF MMP-8 concentrations according to gestational age (GA) at amniocentesis (i.e., <26 weeks, 26–30 weeks, and 30–34 weeks) in PTL (median: 131.0 ng/mL, range [0.3–3929.0 ng/mL] vs. median: 13.1 ng/mL, range [0.3–6142.6 ng/mL] vs. median: 1.4 ng/mL, range [0.3–3116.4 ng/mL]; *p* < 0.001, Kruskal–Wallis test) (**a**) and preterm-PROM (median: 161.0 ng/mL, range [0.3–5019.5 ng/mL] vs. median: 31.0 ng/mL, range [0.3–3171.2 ng/mL] vs. median: 4.5 ng/mL, range [0.3–2313.4 ng/mL]; *p* < 0.001, Kruskal–Wallis test) (**b**).

AF MMP-8 concentrations are continuously and significantly increased with decreasing GA at amniocentesis (i.e., <26 weeks, 26–30 weeks, and 30–34 weeks) in PTL (Figure 2a) and preterm-PROM (Figure 2b).

### 3.4. Relationships between Gestational Age (GA) at Amniocentesis and Amniocentesis-to-Delivery (ATD) Interval in PTL and Preterm-PROM

**Figure 3:**
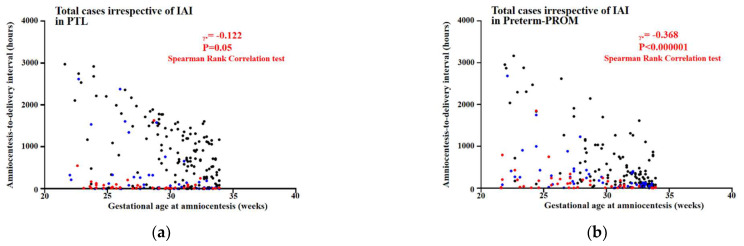
Inverse correlations are shown between gestational age (GA) at amniocentesis and amniocentesis-to-delivery (ATD) interval among cases with PTL (**a**) and among those with preterm-PROM (**b**). Black, blue, and red dots indicate cases with inflammatory-free AF, mild IAI and severe IAI, respectively. Correlation coefficients of Spearman’s rank correlation test and *p*-values are shown.

Figure 3 shows inverse correlations between GA at amniocentesis and ATD interval among cases with PTL (Spearman’s rank correlation test, γ = −0.122, *p* = 0.05) and among those with preterm-PROM (Spearman’s rank correlation test, γ = −0.368, *p* < 0.000001) irrespective of the presence and/or intensity of IAI.

### 3.5. Relationships between Gestational Age (GA) at Amniocentesis and Amniocentesis-to-Delivery (ATD) Interval in the Context of the Same Intensity of Intra-Amniotic Inflammation (IAI) in PTL and Preterm-PROM

**Figure 4:**
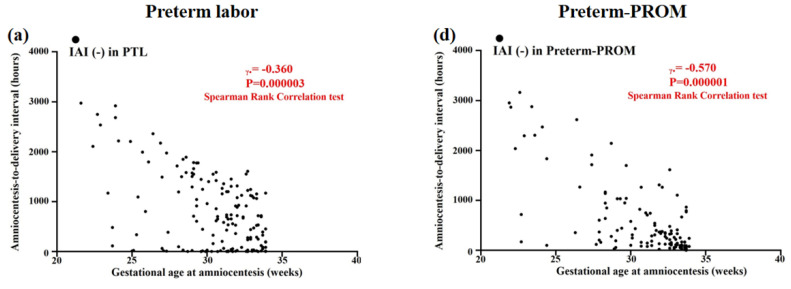

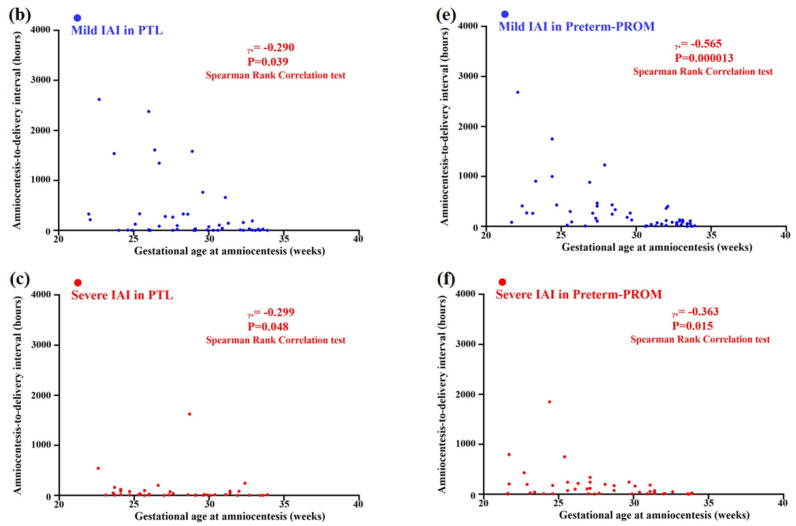
Inverse correlations are shown between gestational age (GA) at amniocentesis and amniocentesis-to-delivery (ATD) interval in the context of the same intensity of intra-amniotic inflammation (IAI) among cases with PTL [i.e., (**a**) intra-amniotic inflammation (IAI) [-] (defined as AF MMP-8 concentration < 23 ng/mL; black dot); (**b**) mild IAI (defined as AF MMP-8 concentration ≥ 23 ng/mL; blue dot); and (**c**) severe IAI (defined as AF MMP-8 concentration ≥ 350 ng/mL; red dot)], and among those with preterm-PROM [(**d**) IAI (-) (black dot); (**e**) mild IAI (blue dot); and (**f**) severe IAI (red dot)]. Correlation coefficients of Spearman’s rank correlation test and *p*-values are shown.

Figure 4 shows inverse correlations between GA at amniocentesis and ATD interval in the context of the same intensity of IAI among cases with PTL (intra-amniotic inflammation [IAI] [-] [Spearman’s rank correlation test, γ = −0.360, *p* = 0.000003] (Figure 4a); mild IAI [Spearman’s rank correlation test, γ = −0.290, *p* = 0.039] (Figure 4b); and severe IAI [Spearman’s rank correlation test, γ = −0.299, *p* = 0.048] (Figure 4c)) and among those with preterm-PROM (IAI [-] [Spearman’s rank correlation test, γ = −0.570, *p* = 0.000001] (Figure 4d); mild IAI [Spearman’s rank correlation test, γ = −0.565, *p* = 0.000013] (Figure 4e); and severe IAI [Spearman’s rank correlation test, γ = −0.363, *p* = 0.015] (Figure 4f)).

## 4. Discussion

### 4.1. The Principal Finding of the Study

**Figure 5:**
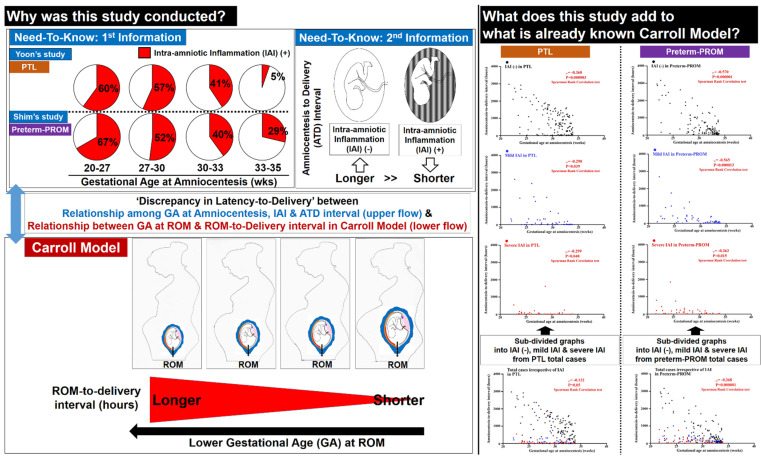
Schema of the limitations of previous studies and the novel findings of the current study. This study originated from ‘the discrepancy of latency-to-delivery’ between GA at amniocentesis through intra-amniotic inflammation (IAI) to latency-to-delivery (the upper flow of left side) and GA at ROM to latency-to-delivery in Carroll model (the lower flow of left side). We demonstrated that PTL and preterm-PROM at a lower GA are associated with a longer latency to delivery even in patients with the same intensity of IAI through the sub-divisional analysis of IAI (the right side). This conceptual model is based on previous reports and data from our study (the upper flow of left side) and Carroll model (the lower flow of left side).

PTL and preterm-PROM at a lower GA are associated with a longer latency to delivery even in patients with the same intensity of IAI (Figure 5). This finding suggests that a more intense IAI may be needed for spontaneous preterm birth at a lower GA.

### 4.2. Limitations of Previous Studies (Figure 6)

**Figure 6:**
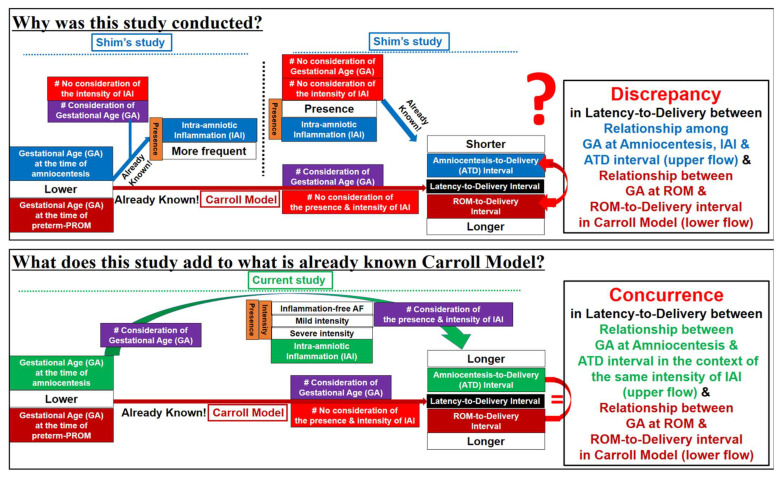
Annotatively transparent description of the limitations of previous studies and the novel findings of current study.

Our previous study by Shim et al. demonstrated that IAI is associated with a significantly shorter latency-to-delivery than inflammation-free AF in preterm-PROM [6]. However, we did not consider GA at preterm-PROM and/or amniocentesis. Moreover, unfortunately, the Carroll model, firstly reporting that the latency-to-delivery from preterm-PROM is longer at a lower GA at the time of ROM, did not analyze the presence and/or intensity of IAI [8]. Based on Shim’s and Carroll’s previous results [6,8], the presence and/or intensity of IAI and GA are critical to the latency-to-delivery from preterm-PROM. Therefore, the presence and/or intensity of IAI should be included in the analysis of the relationship between GA at preterm-PROM and/or amniocentesis and latency-to-delivery. This study is the case (Figure 6).

### 4.3. Why Are PTL and Preterm-PROM at a Lower Gestational Age (GA) Associated with a Longer Latency to Delivery?

The frequency of term birth, preterm birth (PTB) (before 37 weeks), late PTB (between 34 and 37 weeks), and early PTB (before 34 weeks) account for about 90%, 10%, 7%, and 3% of all births, respectively [13]. Moreover, an earlier GA is known to make the induction of labor (IOL) more difficult to succeed in preterm [14] and even term pregnant women [15]. Therefore, one can expect that delivery is more difficult in pregnant women at an earlier GA. Indeed, the levels of oxytocin receptor (OXTR) are known to be one of the contraction-associated proteins (CAPs) (i.e., connexin-43 [CX43], OXTR and prostaglandin [PG] receptors including PGE2EP1 and PGF2αFP) with the function of uterine myometrial activation [16], and are continuously increased with advancing GA in the human placenta [17], decidua [18], and myometrium [18,19]. Based on these findings [13,14,15,16,17,18,19], it is plausible PTL and preterm-PROM at a lower GA are associated with a longer latency to delivery. Indeed, our current study reaffirmed Carroll’s finding reporting the time interval from preterm-PROM to delivery is longer at a lower GA at ROM [8] (Figure 3).

### 4.4. Prostaglandins (PGs) Change in Amniotic Fluid (AF) and Amnion According to the Development of Inflammation and Advancing Gestational Age (GA) Ultimately Leading to Labor and Delivery

PGs are the key activators of the common pathway in human preterm and term labor [20,21,22], which can be explained as follows. Firstly, there is accumulating evidence that PGs in inflammatory AF increase in cases with preterm-PROM [23] and PG production increases in bacteria-simulated amnion cells [24,25]. It should be noted that an increased AF PG F2α (PGF2α) was an independent predictor of impending delivery even after adjusting GA and IAI [23], and an increased PG E2 in amnion cells cultured following spontaneous labor resembled that obtained from amnion cells following addition of filtered bacterial medium [25]. These findings are consistent with Xu’s previous result that PGF2α stimulates all CAPs (i.e., PTGS2 and subsequent PGE2EP1/PGF2αFP, OXTR, and CX43) in human pregnant myometrial smooth muscle cells [22]. Secondly, in the context of term gestation, PGF2α concentrations in AF [26] and PTGS2 mRNA expression in amnion [27] abruptly increase before the onset of labor and continuously increase with advancing gestation from 38 weeks to 40 weeks. These findings correspond with the previous results as in the following: (1) Nielsen et al. demonstrated that the prevalence of unfavorable Bishop scores (≤5) decreased with increasing GA until 41 weeks during term gestation, ultimately leading to an immediate labor and delivery [15]; and (2) Chow et al. found that the levels of CX43 mRNA in myometrial tissue collected from women not in labor significantly increased between 37 and 40 weeks, with a further significant increase occurring during labor [28].

### 4.5. Biologic Plausibility about the Current Study’s Findings That PTL and Preterm-PROM at a Lower Gestational Age (GA) Are Associated with a Longer Latency to Delivery Even in the Context of the Same Intensity of IAI

It is well-known that preterm gestations with a lower GA have a significantly higher frequency of IAI in both PTL and preterm-PROM [4,6]. Moreover, we previously demonstrated the intensity of IAI continuously increases with decreasing GA during preterm gestation even in the context of the same severity of acute chorioamnionitis [29]. These findings suggest that a more intense IAI should be required for the preterm delivery at a lower GA. Based on the relationship among GA, IAI, AF PGs, and impending preterm delivery in previous studies [4,6,23,29], a higher amount of PGs recruitment into AF would be expected in preterm birth at a lower GA. Notably, OXTR is known to be much lower with an earlier GA in the human gestational tissues (i.e., placenta [17], decidua [18], and myometrium [18,19]). Therefore, it is plausible that a more intense IAI for the recruitment of higher PG concentrations should be required in preterm birth at a lower GA. Moreover, one can expect that preterm birth at a lower GA is much rarer in the context of same intensity of IAI. Indeed, our current study shows PTL and preterm-PROM at a lower GA are associated with a longer latency to delivery in the context of the same intensity of IAI.

### 4.6. Major Strengths and Weakness of Current Study

This study had a couple of major strengths. Firstly, the study population is a very large cohort of singleton pregnancies (476 cases; PTL, 253 cases and preterm-PROM, 223 cases) with AF obtained between 21.6 and 33.9 weeks. Secondly, we examined the relationship between GA at amniocentesis and latency-to-delivery according to the same intensity of IAI (i.e., inflammation-free AF, mild IAI, and severe IAI), because the presence and/or intensity of IAI is very critical to the latency-to-delivery in both PTL and preterm-PROM [4,6]. Therefore, each patient could be comparable according to GA at amniocentesis when latency-to-delivery was analyzed after the division by the intensity of IAI. The potential weaknesses of this study are as follows. Firstly, it did not include cases with GA at amniocentesis beyond 34 weeks in PTL and preterm-PROM. However, a substantial portion of patients with either PTL or preterm-PROM beyond 34 weeks did not have AF results because we did not generally recommend the amniocentesis for the evaluation of IAI and/or fetal lung maturity to these patients. Secondly, the follow-up of neonatal outcomes is missing in the current study, although the information of GA at delivery and birth weight is provided. However, the analysis of neonatal outcomes is beyond the current study’s scope, which is about ‘the analysis of amniocentesis-to-delivery interval’. Thirdly, we looked at latency between amniocentesis and delivery, but not latency between onset of either PTL or preterm-PROM and delivery. However, it was impossible to obtain the exact onset of either PTL or preterm-PROM in all 476 members of the study population, which underwent amniocentesis in the context of retrospective cohort study even through the meticulous review of medical record, because it was very difficult to define the precise time at which PTL or preterm-PROM developed. Moreover, the main objective in our current study is to examine whether either PTL or preterm-PROM at a lower GA is associated with a shorter or longer latency to delivery in cases with the same intensity of IAI. Therefore, we should examine amniotic fluid (AF) inflammatory status at the specific time of GA at amniocentesis, but not either PTL onset or preterm-PROM, for the analysis of latency-to-delivery. Furthermore, Kim BJ et al. previously demonstrated there is no difference in the frequency of IAI among 6~24 h, 24~48 h, 48~72 h, and more than 72 h of ROM-to-amniocentesis interval (that is, the time elapsed between ROM and amniocentesis) after the first 6 h of ROM in preterm-PROM (<35 weeks) [30]. Therefore, we do not believe it is a major source of bias that we looked at latency between amniocentesis and delivery, but not latency between onset of either PTL or preterm-PROM and delivery. Fourthly, it is to include only cases that underwent vaginal delivery, but not Cesarean section (C/S), for the exact investigation of the latency between amniocentesis and spontaneous parturition in cases with PTL and preterm-PROM. However, we included cases that underwent both vaginal and Cesarean delivery in the current study. Nevertheless, we do not believe it is a major source of bias that C/S cases were included in our current manuscript for the following reasons; (1) our institute performed C/S only in the cases with obstetric indications (i.e., labor progression or fetal malpresentation in cases with previous C/S or uterine surgery, placenta previa and/or vaginal bleeding, failure to progress in labor, and fetal distress), and there was no significant difference in the frequency of C/S among group-1 (GA at amniocentesis < 26 weeks), group-2 (GA at amniocentesis 26~30 weeks) and group-3 (GA at amniocentesis 30~34 weeks) in the same intensity of IAI (i.e., IAI [-], mild IAI, and severe IAI) in both PTL and preterm-PROM cases (Table 1 and Table 2); and (2) our data showing inverse correlations between GA at amniocentesis and ATD interval among cases with PTL and among those with preterm-PROM irrespective of the presence and/or intensity of IAI (Figure 3) are very similar to those reported in Carroll’s previous study, although it did not provide any information about the mode of delivery [8].

### 4.7. Clinical Implications of Current Study

To the best of our knowledge, this is the first study reporting PTL and preterm-PROM at a lower GA are associated with a longer latency-to-delivery even in patients with the same intensity of IAI. We reaffirmed the “Carroll-model” about the relationship between GA at preterm-PROM and latency-to-delivery with no consideration of the presence and intensity of IAI [8]. This finding may provide the solid evidence that we can spend more time on the treatment against IAI in both PTL and preterm-PROM cases with a lower GA. Therefore, we should make more effort for the inhibition of IAI and/or acute chorioamnionitis leading to the reduction of neonatal infectious/inflammatory morbidity in both PTL and preterm-PROM cases with a lower GA.

### 4.8. Unanswered Questions and Proposals for Further Studies

The current study may drive other research centers to study the different molecular targets in AF and placenta according to GA even in the context of the same intensity of IAI. This further research is likely to establish the different strategies for the inhibition of spontaneous preterm birth according to GA. Moreover, other future studies including animal experiments should be investigated to determine whether neonatal outcomes such as CNS injury are improved or aggravated in cases with the prolongation of GA, even using antibiotic treatment in the context of severe IAI.

## 5. Conclusions

PTL and preterm-PROM at a lower GA are associated with a longer latency to delivery even in patients with the same intensity of IAI.

## Figures and Tables

**Table 1:** Clinical characteristics and pregnancy outcomes according to gestational age at amniocentesis in PTL cases with the same intensity of intra-amniotic inflammation (IAI).

GA at Amniocentesis	<26 Wks Group-1	26 Wks~30 Wks Group-2	30 Wks~34 Wks Group-3	*p* ^†^
	(*n* = 42)	(*n* = 76)	(*n* = 135)	
IAI (-) [*n* = 158]	(*n* = 17)	(*n* = 40)	(*n* = 101)	
Maternal age, years (mean ± SD)	31.53 ± 4.32	32.03 ± 5.05	30.37 ± 4.13	NS (0.169)
Nulliparity	64.7% (11/17)	57.5% (23/40)	53.5% (54/101)	NS (0.665)
GA at delivery, wks (mean ± SD)	33.42 ± 5.99	34.58 ± 4.50	35.51 ± 2.90	NS (0.539)
Birth weight, g (mean ± SD)	2240 ± 987	2384 ± 859	2517 ± 677	NS (0.642)
Male newborn	47.1% (8/17)	55.0% (22/40)	50.5% (51/101)	NS (0.832)
Cesarean section	29.4% (5/17)	55.0% (22/40)	45.5% (46/101)	NS (0.440)
Acute-HCA * or funisitis	35.3% (6/17)	27.5% (11/40)	21.8% (22/101)	NS (0.439)
Mild IAI [*n* = 51]	(*n* = 9)	(*n* = 21)	(*n* = 21)	
Maternal age, years (mean ± SD)	29.56 ± 3.09	29.48 ± 4.51	30.71 ± 4.17	NS (0.578)
Nulliparity	66.7% (6/9)	47.6% (10/21)	47.6% (10/21)	NS (0.584)
GA at delivery, wks (mean ± SD)	27.29 ± 5.04	30.43 ± 3.95	32.50 ± 1.39	<0.001
Birth weight, g (mean ± SD)	1199 ± 829	1655 ± 810	1966 ± 389	0.006
Male newborn	66.7% (6/9)	57.1% (12/21)	38.1% (8/21)	NS (0.272)
Cesarean section	33.3% (3/9)	47.6% (10/21)	38.1% (8/21)	NS (0.715)
Acute-HCA * or funisitis	66.7% (6/9)	71.4% (15/21)	61.9% (13/21)	NS (0.807)
Severe IAI [*n* = 44]	(*n* = 16)	(*n* = 15)	(*n* = 13)	
Maternal age, years (mean ± SD)	31.69 ± 4.60	29.80 ± 3.80	30.54 ± 5.13	NS (0.707)
Nulliparity	37.5% (6/16)	40.0% (6/15)	38.5% (5/13)	NS (0.990)
GA at delivery, wks (mean ± SD)	25.01 ± 0.91	28.92 ± 2.89	32.20 ± 1.28	<0.001
Birth weight, g (mean ± SD)	759 ± 167	1286 ± 468	1771 ± 553	<0.001
Male newborn	43.8% (7/16)	53.3% (8/15)	69.2% (9/13)	NS (0.388)
Cesarean section	37.5% (6/16)	20.0% (3/15)	38.5% (5/13)	NS (0.480)
Acute-HCA * or funisitis	87.5% (14/16)	93.3% (14/15)	84.6% (11/13)	NS (0.757)

^†^ Among three groups, Kruskal–Wallis test was used for comparison of continuous variables and Pearson’s chi-square test was used for comparison of the proportions. *NS*, not significant; *GA*, gestational age; *AF*, amniotic fluid; *PTL*, preterm labor and intact membranes; *acute-HCA*, acute histologic chorioamnionitis. * Means the presence of chorio-deciduitis, amnionitis, or chorionic plate inflammation.

**Table 2:** Clinical characteristics and pregnancy outcomes according to gestational age at amniocentesis in preterm-PROM cases with the same intensity of intra-amniotic inflammation (IAI).

GA at Amniocentesis	<26 Wks Group-1	26 Wks~30 Wks Group-2	30 Wks~34 Wks Group-3	*p* ^†^
	(*n* = 39)	(*n* = 56)	(*n* = 128)	
IAI (-) [*n* = 127]	(*n* = 12)	(*n* = 28)	(*n* = 87)	
Maternal age, years (mean ± SD)	29.83 ± 2.59	30.29 ± 5.26	30.84 ± 4.53	NS (0.573)
Nulliparity	83.3% (10/12)	57.1% (16/28)	51.7% (45/87)	NS (0.117)
GA at delivery, wks (mean ± SD)	35.00 ± 6.25	33.37 ± 4.02	34.32 ± 2.00	NS (0.071)
Birth weight, g (mean ± SD)	2581 ± 1344	2047 ± 767	2287 ± 485	0.027
Male newborn	50.0% (6/12)	60.7% (17/28)	58.6% (51/87)	NS (0.814)
Cesarean section	50.0% (6/12)	35.7% (10/28)	34.5% (30/87)	NS (0.576)
Acute-HCA * or funisitis	41.7% (5/12)	46.4% (13/28)	44.8% (39/87)	NS (0.962)
Mild IAI [*n* = 52]	(*n* = 12)	(*n* = 14)	(*n* = 26)	
Maternal age, years (mean ± SD)	33.25 ± 3.89	30.07 ± 4.20	29.92 ± 3.67	NS (0.075)
Nulliparity	16.7% (2/12)	21.4% (3/14)	53.8% (14/26)	0.034
GA at delivery, wks (mean ± SD)	27.87 ± 4.58	30.16 ± 2.03	33.01 ± 1.10	<0.001
Birth weight, g (mean ± SD)	1234 ± 792	1500 ± 514	1957 ± 257	<0.001
Male newborn	66.7% (8/12)	42.9% (6/14)	53.8% (14/26)	NS (0.479)
Cesarean section	33.3% (4/12)	42.9% (6/14)	50.0% (13/26)	NS (0.625)
Acute-HCA * or funisitis	75.0% (9/12)	92.9% (13/14)	84.6% (22/26)	NS (0.453)
Severe IAI [*n* = 44]	(*n* = 15)	(*n* = 14)	(*n* = 15)	
Maternal age, years (mean ± SD)	31.67 ± 4.62	31.86 ± 5.60	31.13 ± 3.40	NS (0.987)
Nulliparity	46.7% (7/15)	35.7% (5/14)	40.0% (6/15)	NS (0.832)
GA at delivery, wks (mean ± SD)	25.47 ± 3.51	28.48 ± 1.27	32.27 ± 1.13	<0.001
Birth weight, g (mean ± SD)	914 ± 730	1208 ± 182	1892 ± 308	<0.001
Male newborn	73.3% (11/15)	71.4% (10/14)	53.3% (8/15)	NS (0.446)
Cesarean section	26.7% (4/15)	57.1% (8/14)	26.7% (4/15)	NS (0.147)
Acute-HCA * or funisitis	86.7% (13/15)	78.6% (11/14)	100% (15/15)	NS (0.184)

^†^ Among three groups, Kruskal–Wallis test was used for comparison of continuous variables and Pearson’s chi-square test was used for comparison of the proportions. *NS*, not significant; *GA*, gestational age; *AF*, amniotic fluid; *preterm-PROM*, preterm premature rupture of membranes; *acute-HCA*, acute histologic chorioamnionitis. * Means the presence of chorio-deciduitis, amnionitis, or chorionic plate inflammation.
